# A study of the effect of sports participation on married youth’s fertility intentions - chain mediation based on marital and family functioning

**DOI:** 10.3389/fpubh.2025.1573613

**Published:** 2025-07-18

**Authors:** Li Lin, Xinze Li, Cen Cong

**Affiliations:** ^1^School of Physical Education, Jimei University, Xiamen, China; ^2^School of Humanities, Beijing Sport University, Beijing, China; ^3^College of Physical Education and Health, East China Jiaotong University, Nanchang, China

**Keywords:** sports participation, fertility intention, family function, marital function, self-determination theory, family systems theory

## Abstract

**Introduction:**

This study investigates the impact of sports participation on the fertility intentions of married young adults in China, utilizing data from the 2022 China Family Panel Studies (CFPS). By integrating Self-Determination Theory (SDT) and Family Systems Theory (FST), the study hypothesizes that sports participation enhances fertility intentions through mediation by marital and family functioning.

**Methods:**

A sample of 1,087 valid cases was analyzed using probit regression and structural equation modeling.

**Results:**

The results indicate that each one-unit increase in sports participation is associated with a 0.048 higher probability of intending to have a child. Specifically, Structural equation modeling with 1,000 bootstrapped samples confirms that marital functioning mediates this relationship (indirect effect = 0.023, 13.7% of the total effect; *p* < 0.10), family functioning mediates more strongly (indirect effect = 0.082, 48.8%; *p* < 0.01), and the chain mediation through both subsystems accounts for 0.063 (37.5%; *p* < 0.01).

**Discussion:**

This study underscores the importance of both marital and family subsystems in shaping fertility decisions, suggesting that policies promoting sports participation and family-centered support may effectively enhance fertility intentions among young married individuals.

## 1 Introduction

The global decline in fertility rates, particularly among young adults, presents significant challenges for aging societies, with China exemplifying this demographic crisis. By 2020, 13.5% of China’s population was aged 65 or older, a proportion projected to double by 2050. Meanwhile, the total fertility rate has stagnated at 1.3, well below replacement levels. Despite aggressive pronatalist policies, including the 2021 “universal three-child” initiative, birth rates remain persistently low. Traditional explanations focusing on economic barriers, such as rising childcare costs and career trade-offs, do not fully account for this trend, as even affluent cohorts show suppressed fertility intentions (1). This paradox highlights the critical role of non-economic factors in shaping reproductive behaviors, a dimension that remains underexplored in current research.

Self-Determination Theory (SDT) provides a robust framework to address this gap. Centered around the fulfillment of three innate psychological needs—autonomy (volitional choice), competence (mastery of challenges), and relatedness (social connectedness)—SDT asserts that intrinsic motivation, rather than external incentives, drives sustained behavioral engagement (2). When applied to fertility decisions, SDT suggests that young adults are more likely to pursue parenthood when they perceive it as a self-determined goal, feel capable of managing the challenges of parenting, and have strong familial support (3). Sports participation, as a voluntary and socially embedded activity, addresses these needs uniquely (4). For example, couples who engage in shared physical activities, such as cycling or yoga, often report increased emotional synchrony and collaborative decision-making, thereby enhancing their perceived readiness for parenting.

However, the fertility-enhancing effects of sports participation extend beyond individual psychology. Family Systems Theory (FST) posits that reproductive choices are shaped by interdependent subsystems: the marital subsystem, which governs dyadic interactions, and the family subsystem, which encompasses intergenerational cohesion and collective resilience (5, 6). In China’s collectivist context, marital harmony not only strengthens spousal bonds but also signals familial stability to extended family members, thereby activating intergenerational support networks that are crucial for childcare—a resource that has been shown to enhance fertility intentions (7). Sports participation amplifies this dynamic: joint physical activities promote conflict resolution and stress resilience, while family fitness rituals (e.g., multigenerational hiking) help cultivate a shared identity, reframing parenthood as an extension of familial legacy rather than a personal sacrifice (8).

Despite this potential, existing studies remain fragmented, focusing either on individual psychological outcomes or static family variables, while neglecting the hierarchical interplay between marital and familial subsystems. Moreover, the cultural specificity of these mechanisms—particularly in East Asian societies where filial piety and intergenerational reciprocity influence fertility decisions—has been insufficiently explored (9). For instance, the involvement of grandparents in childcare, which alleviates parenting stress in urban Chinese families, often depends on observed marital cohesion—an aspect that has not been adequately addressed in Western-centric models.

This study addresses these gaps by proposing an integrated SDT-FST systems framework. We hypothesize that sports participation enhances fertility intentions through a sequential mediation process: voluntary physical activity first strengthens marital subsystem functioning by fulfilling autonomy, competence, and relatedness needs, which then activates family subsystem resources (e.g., intergenerational support), ultimately fostering a socio-psychological environment conducive to childbearing. By elucidating this interconnected mechanism, we aim to shift the paradigm from “economic determinism” to “relational resilience” in fertility research, providing policymakers with actionable strategies to strengthen pronatalist policies through culturally sensitive, family-centered interventions.

## 2 Literature review and research hypothesis

### 2.1 Sports participation and fertility intention

Self-Determination Theory (SDT) posits that human motivation and well-being are influenced by the fulfillment of three innate psychological needs: autonomy (volitional control over actions), competence (perceived mastery in goal-directed behaviors), and relatedness (meaningful social connections). We propose that sports participation provides a behavioral context for satisfying these needs, thereby fostering a psychological predisposition toward childbearing among young adults.

First, sports participation, as a self-chosen activity (fulfilling the autonomy need), enhances individuals’ sense of agency in life planning. When young people voluntarily engage in sports, they internalize the belief that major life decisions—such as parenthood—can be actively managed (10). For example, prioritizing regular exercise reflects self-determination, which may extend to proactive family planning.

Second, the physiological and psychological benefits of sports participation (e.g., stress resilience through endorphin release, mood improvement via serotonin regulation) directly enhance perceived competence to manage parenting challenges (11). Enhanced physical fitness and mental stamina from sports engagement foster confidence in handling the demands of childcare, aligning with SDT’s emphasis on mastery experiences.

Third, sports participation facilitates social connectedness through team-based interactions or shared activities with partners, thereby strengthening perceived support networks that are critical for future childcare (12). Joint participation in sports, such as couple yoga or family cycling, nurtures relational bonds and reinforces the social infrastructure necessary for parenting.

Critically, these SDT-driven mechanisms may mitigate contemporary barriers to fertility. While economic constraints and digital overload erode confidence in parenting, sports participation-induced need satisfaction could provide compensatory motivational resources. Supporting this, longitudinal evidence links regular sports engagement to an 18–24% higher likelihood of transitioning to parenthood within 5 years, independent of income and education (13). Accordingly, this paper proposes research hypothesis 1:

*H1*: The higher the level of sports participation among young people, the stronger their desire to have children.

### 2.2 Mechanism

The intricate relationship between sports participation and young adults’ fertility intentions is best understood through a multilevel lens of family system functioning—a dynamic construct that bridges intimate dyadic interactions and broader familial ecosystems. Drawing on Family Systems Theory (FST), which emphasizes the interdependence of subsystems within families, this framework posits that sports activities serve as a catalyst for enhancing fertility intentions by optimizing two key subsystems: the marital subsystem and the family subsystem.

At the marital level, sports participation fosters relational resilience by addressing core psychological needs identified in Self-Determination Theory (SDT). Voluntary engagement in physical activities allows couples to exercise shared agency in selecting routines that align with their values (14), such as morning runs or weekend yoga sessions. This volitional choice translates into greater collaborative decision-making in other life domains, including family planning. Regular exercise also enhances physiological and psychological resilience, reducing cortisol levels and improving stress management capacities, which are critical for navigating parenting challenges (15). Furthermore, synchronized physical activities, such as partner dancing or doubles tennis, elevate oxytocin levels, fostering emotional attunement and creating a feedback loop of intimacy and mutual support. These marital enhancements extend beyond the dyad, positively influencing the broader family subsystem.

The family subsystem encompasses intergenerational solidarity, collective resource-sharing, and shared identity formation. In collectivist societies like China, marital harmony acts as a social signal to extended family members, particularly grandparents, who view cohesive spousal relationships as indicative of familial stability. This perception increases their willingness to provide childcare support, thereby alleviating the economic and logistical burdens of childrearing. Family sports rituals, such as intergenerational hiking trips or multigenerational fitness challenges, cultivate a culture of collective problem-solving, reinforcing perceived parenting efficacy (16). Shared athletic achievements, like completing a community marathon as a family unit, solidify a “we-identity,” reframing parenthood as an extension of familial purpose and legacy (17).

The cultural embeddedness of this mechanism is crucial. In individualistic Western contexts, familial resilience often relies on nuclear family resources and institutional support, whereas in collectivist East Asian societies, intergenerational networks play a vital role. For instance, a Chinese couple’s participation in tai chi sessions with older adult parents may simultaneously enhance marital cohesion, demonstrate filial piety, and reinforce a collective identity centered on health and longevity (32)—all synergistically elevating fertility intentions. This highlights the necessity of culturally nuanced theoretical models. Accordingly, this paper proposes research hypotheses 2 and 3:

*H2*: Sports participation can promote youth fertility intentions by optimizing family system functioning.

*H2a*: Sport participation can promote youth fertility intentions by optimizing marital functioning.

*H2b*: Sports participation can promote youth fertility intentions by optimizing family functioning.

*H2c*: Sports participation can increase youth fertility intentions through the interaction of marital and family functioning.

*H3*: Optimization of marital functioning can promote family functioning.

## 3 Methodology and data

### 3.1 Data sources

This study utilizes data from the China Family Panel Studies (CFPS), which covers 31 provinces, autonomous regions, and municipalities directly under the central government in China, and includes information on social communities, families, and individuals at multiple levels, making it geographically broad and representative. Given the ongoing epidemic in China in 2020, the fertility intentions of young and middle-aged individuals are expected to be influenced by the pandemic. The study is based on CFPS 2022 data, with a detailed preprocessing procedure. In accordance with the criteria set by the World Health Organization (WHO), China’s tradition of “marriage before childbearing,” and the legal minimum marriage age, the study focuses on “married young people,” specifically male respondents aged 22 to 45 and female respondents aged 20 to 40. After excluding missing values, extreme values, and outliers, a final sample of 1,087 valid cases was obtained. The basic characteristics of the sample are shown in Table 1, which includes an equal proportion of male and female respondents, with 33.06% of the total sample coming from the eastern region. The age distribution of the respondents is balanced, and 47.59% of participants had received higher education.

**Table 1:** Basic characteristics of the sample.

Variable	Sort	Frequency	Scale
Sex	Male	537	49.39%
	Female	550	50.61%
District	The East	429	33.06%
	Middle part	283	26.35%
	West	294	27.55%
	Northeast part	131	13.04%
Age	20 to 30 Years old	521	48.10%
	30 to 45 Years old	566	49.61%
Educational level	Uneducated	175	16.54%
	Below primary school	207	19.43%
	Middle school and high school	188	16.46%
	College or above	517	47.59%

### 3.2 Description of variables

#### 3.2.1 Explained variables

Fertility intention (Fer) is the explanatory variable (shown in Table 2). Referring to the practice of related studies to the CFPS2022: “Will you have a child in the next two years?” The data from the question “Will you have a child in the next two years?” was used to measure Fer, with values ranging from 0 and 1, with 0 being no and 1 being yes.

**Table 2:** Variable settings and descriptions.

Variable type	Variable name	Variable symbol	Variable declaration
Explained variable	Fertility intention	Fer	Will there be a desire to have children in the next 2 years, with 0 being no and 1 being yes
Explanatory variable	Sports participation	Spo	Never = 0; less than 1 time per month on average = 1; more than 1 time per month on average but less than 1 time per week = 2; 1–2 times per week on average = 3; 3–4 times per week on average = 4; 5 times per week and more on average = 5; 1 time per day = 6; twice a day and more = 7
Intermediate variable	Marital functioning	Mar	How satisfied are you with your current marriage: Very dissatisfied = 1; Quite dissatisfied = 2; Fair = 3; Quite satisfied = 4; Very satisfied = 5
		Mai	How satisfied are you with your spouse’s financial income: Very dissatisfied = 1; Quite dissatisfied = 2; Fair = 3; Quite satisfied = 4; Very satisfied = 5
		Mad	How satisfied are you with your spouse’s contribution to the family: Very dissatisfied = 1; Quite dissatisfied = 2; Fair = 3; Quite satisfied = 4; Very satisfied = 5
	Family functioning	Hap	Level of happiness of family members: very unhappy = 1; relatively unhappy = 2; average = 3; relatively happy = 4; very happy = 5
		Has	Frequency of intergenerational meetings: number of days per week with elders/children, taking values from 0 to 7
		Haw	Intergenerational status: trust in parents/children: values from 0 to 10
Control variable	Sex	Gen	Female = 0; Male = 1
	Age	Age	Year of survey minus year of birth
	Number of marriage segments	Mag	Number of marriages, 1 for one marriage, 2 for another marriage after divorce
	Number of cohabitation segments	Coh	Frequency of cohabitation with different members of the opposite sex
	Level of leisure	Qgq	The longer the working hours, the lower the level of leisure, taking the logarithm of the working hours.
	Security situation	Qia	0 for not having any social security contributions, 1 otherwise
	Age of first marriage	Maa	Year of first marriage - Year of birth
	Educational level	Edu	Illiterate/semi-literate = 1; Primary school = 2; Junior high school = 3; Senior high school/secondary/technical/vocational school = 4; College = 5; Bachelor’s degree = 6; Master’s degree = 7; Doctorate = 8
	Health status	Hea	Very Unhealthy = 1; Less Healthy = 2; Generally = 3; Relatively Healthy = 4; Very Healthy = 5
	Family economic income	Inc	Total family income
	Number of family members	Num	Number of current members of the household
	Gross regional product *per capita*	Cap	Natural logarithm of gdp *per capita* of the province (Region, City) of the respondent

#### 3.2.2 Explanatory variables

Sports participation (Spo) was the core explanatory variable (shown in Table 2) and was measured with data from the CFPS2022, “How often did you/do you participate in sports, fitness, and recreational activities in the past 12 months?” Data from one question was measured. Specifically, “less than 1 time per month on average” “more than 1 time per month on average, but less than 1 time per week” “1–2 times per week on average” “on average 3–4 times per week” ‘5 times and more per week on average’ ‘1 time per day’ ‘twice a day and more’, corresponding to values from 1 to 7, respectively. “Never attend” is assigned a value of 0.

Mediating explanatory variables include family functioning and marital functioning (as shown in Table 2). Marital functioning includes marital satisfaction (Mar), spousal income satisfaction (Mai), and spousal giving satisfaction (Mad), using a range of values from 1 to 5. Family functioning included family happiness (Hap), using a range of values from 1 to 5. Intergenerational meeting frequency (Has), using a range of values from 1 to 7. Intergenerational relationship status (Haw), using a range of values from 1 to 10.

#### 3.2.3 Control variables

Referring to the research results of scholars (18–20), this paper selected nine variables as control variables (as shown in Table 2). Among them, personal factors include gender (Gen), age (Age), education (Edu), self-rated health (Hea), leisure (Qgq), and security (Qia), marital status includes age at first marriage (Maa), number of marriage segments (Mag), and number of cohabitation segments (Coh), and family factors include total annual household income (Inc) and number of household members (Num).

### 3.3 Model construction

In order to analyze the effect of sports participation on the fertility intentions of married youth. As shown in Equation (1), the study constructed a binary probit regression analysis using Fer as the dependent variable with the model equation:


(1) $$\text{Probit}(p)=[\beta_{0}+\beta_{1}\mathrm{Spo}+\beta_{2}\text{Contro}l_{1}+\ldots +\beta_{p}{\text{Control}}_{p}]+\epsilon_{1}$$

Where p represents the probability that Fer is 1, which indicates that the individual has a desire to have children, is the individual’s level of sports participation, is a control variable, and is a random error term.

In order to clarify the micro role of sports participation in influencing the fertility intentions of married youth, the study attempts to apply structural equation modeling to decompose the mediating effect. Compared to the stepwise method, which has traditionally been used as a way to test and estimate the mediating effects, Structural Equation Model (SEM) can handle multiple dependent variables at the same time and conduct path tests when there are mediating variables family functioning and marital functioning. The specific formula is:


(2) η=βη+Γξ+ξ

(3) $$Y=\Lambda_{Y}\eta +\delta$$

(4) $$X=\Lambda_{X}\eta +\varepsilon \;$$

Equation 2 is a structural equation reflecting the relationship between exogenous latent variables, endogenous latent variables and exogenous explicit indicators, where β is the path coefficient, which represents the relationship between endogenous latent variables, and Γ is the path coefficient, which represents the effect of exogenous explicit indicators on endogenous latent variables. Equations 3, 4 are measurement equations, which represent the relationship between potential variables and explicit indicators, where $\Lambda_{Y}$ and $\Lambda_{X}$ represent the relationship between exogenous potential variables and endogenous potential variables and explicit indicators, respectively, and ξ , δ and ε are error terms. The statistics and analysis of the above data were performed using the software Stata 16.0.

## 4 Results

### 4.1 Descriptive statistics and correlation analysis

The valid sample of this study was 1,087, and the mean, standard deviation, minimum and maximum values of the variables and the correlation coefficients are shown in Tables 3, 4. Sports participation (Spo) and fertility intention (Fer), marital functioning (Mar, Mai, Mad) and fertility intention (Fer), and family functioning (Hap, Has, Haw) and fertility intention (Fer) are significantly correlated, and all the correlation coefficients are less than 0.7, and the maximum of the variance inflation factor VIF for each variable is less than 5, which indicates that there is no problem of multiple covariance among factors.

**Table 3:** Table of descriptive statistics.

Variable name	Mean	SD	Min	Max
Fertility intention	0.125	0.331	0	1
Sports participation	1.672	2.386	0	7
Marital function Mar	4.418	0.891	1	5
Marriage function Mai	4.101	1.156	1	5
Marriage function Mad	4.235	1.021	1	5
Family features Hap	4.067	0.818	1	5
Family features Has	5.121	3.622	0	7
Family features Haw	8.961	2.849	0	10
Sex	0.494	0.500	0	1
Age	35.153	14.044	20	45
Number of marriage segments	0.999	0.050	1	2
Number of cohabitation segments	0.020	0.140	0	2
Level of leisure	35.474	29.716	0	168
Security situation	0.749	0.566	0	1
Age of first marriage	24.243	5.341	20	45
Educational level	3.046	1.459	1	8
Health status	3.063	1.175	1	5
Family economic income	10.416	1.560	0	13.816
Number of family members	4.239	1.998	1	16
Gross regional product *per capita*	2.484	1.209	1	4

**Table 4:** Matrix of correlation coefficients.

Variable name	Fer	Spo	Gen	Age	Hea	Maa	Edu
Fer	1						
Spo	0.031***	1					
Gen	0.067***	0.038***	1				
Age	−0.326***	0.136***	0.092***	1			
Hea	0.057***	0.005	0.065***	−0.242***	1		
Maa	0.095***	0.091***	0.182***	0.038**	0.007	1	
Edu	0.203***	0.179***	0.087***	−0.439***	0.106***	0.198***	1
Num	−0.127***	−0.075***	−0.016**	−0.165***	0.050***	−0.069***	−0.063***
Cap	−0.019*	−0.011	0.012	−0.042***	−0.012	−0.013	−0.104***
Inc	0.055***	0.069***	0.163***	−0.149***	0.047***	0.128***	0.269***
Coh	0.267***	−0.005	0.016**	−0.182***	0.033***	0	0.134***
Mag	−0.013	0.012	−0.013	−0.003	−0.016**	0.034**	0.008
Qgq	0.014	−0.158***	0.204***	−0.325***	0.129***	0.004	0.091***
Qia	0.017	−0.163***	−0.007	−0.632***	0.157***	−0.024	0.183***
Hap	0.063***	0.076***	0.043***	0.018**	0.212***	−0.014	0.051***
Has	0.017**	0.041***	−0.099***	0.107***	−0.050***	−0.066***	−0.040***
Haw	0.018*	0.022***	0.01	−0.116***	0.120***	−0.034**	0.113***
Mar	0.051***	0.034***	0.187***	0.040***	0.147***	0.003	−0.017**
Mai	0.054***	0.023***	0.153***	0.011	0.149***	−0.011	0.007
Mad	0.031***	0.015*	0.332***	0.143***	0.100***	0.013	−0.081***

Variable name	Num	Cap	Inc	Coh	Mag	Qgq	Qia
Num	1						
Cap	0.186***	1					
Inc	−0.039***	0.004	1				
Coh	−0.064***	−0.007	0.048***	1			
Mag	0.004	−0.002	−0.025**	−0.076***	1		
Qgq	0.035***	0.066***	0.052***	0.033***	0.006	1	
Qia	0.115***	0.044***	0.141***	0.062***	0.014*	0.346***	1
Hap	−0.006	−0.076***	0.037***	0.008	0	−0.024***	−0.040***
Has	0.129***	−0.011	−0.077***	−0.025***	0.049***	−0.094***	−0.004
Haw	0	−0.041***	0.048***	0.012	0.047***	0.146***	0.215***
Mar	0.001	−0.032***	0.023*	0.014*	0.007	0.029***	−0.015**
Mai	−0.015*	−0.047***	0.028**	0.007	0.003	0.006	0
Mad	−0.014*	−0.007	0.030**	−0.015*	0	0.045***	−0.056***

Variable name	Hap	Has	Haw	Mar	Mai	Mad
Hap	1					
Has	0.062***	1				
Haw	0.191***	0.410***	1			
Mar	0.375***	0.035***	0.070***	1		
Mai	0.342***	0.047***	0.081***	0.573***	1	
Mad	0.288***	0.011	0.037***	0.507***	0.528***	1

*p < 0.1 **p < 0.05 ***p < 0.01, the following table is the same.

### 4.2 Results of baseline regression and path analysis

#### 4.2.1 Baseline regression

To assess the specific impact of sports participation on the fertility intentions of married youth, two probit models were constructed for this study. The results are shown in Table 5. Model (1), which does not include any control variables, shows a statistically significant coefficient of 0.025 for sports participation (*p* < 0.01). When relevant control variables are introduced, Model (2) reveals a slight increase in the coefficient to 0.048, which is significant at the 0.1 level. This suggests that each unit increase in sports participation is associated with an average increase of 0.048 units in the fertility intentions of married youth. Both Models (1) and (2) indicate a significant positive effect of sports participation on the fertility intentions of married youth, thereby supporting the verification of hypothesis H1.

**Table 5:** Regression results of sports participation on young people’s fertility intentions.

Variable name	Ols (1)	Ols (2)
	Fertility intention	Fertility intention
Sports participation	0.248*** (2.81)	0.048* (1.69)
Sex		0.252*** (2.13)
Age		−0.112*** (−8.27)
Number of marriage segments		−1.466 (−1.17)
Number of cohabitation segments		2.552*** (3.42)
Level of leisure		0.003 (0.82)
Security situation		0.118 (1.23)
Age of first marriage		0.089*** (5.50)
Educational level		0.050 (1.04)
Health status		0.077 (1.32)
Family economic income		0.002 (0.05)
Number of family members		−0.054** (−1.90)
Gross regional product *per capita*		−0.019 (−0.51)
Constant term	−1.184*** (−52.90)	1.364** (1.93)
LR chi^2^	7.79	157.88
*N*	1,087	1,087
Adj.R^2^	0.042	0.141

#### 4.2.2 Path analysis

The structural equation model was employed to verify the pathways through which sports participation influences the fertility intentions of married young individuals, primarily by comparing the expected covariance matrix of the hypothesized model with the actual covariance matrix of the sample data. Specifically, the model fit indices are as follows: χ^2^/df = 0.010 (< 3), RMR = 0.031 (< 0.050), CFI = 0.915 (> 0.900), and GFI = 0.922 (> 0.900). These results suggest that the hypothesized model fits the data well, making it suitable for subsequent path analysis.

In this study, the mediating effect was tested using the Bootstrap method with 1,000 samples, and the results were bias-corrected with confidence intervals set at 95%. As shown in Tables 6, 7, the total mediating effect of family system functionality in the relationship between sports participation and fertility intentions was 0.168. Specifically, the mediating effect of marital functionality on the relationship between sports participation and fertility intentions was 0.023, while the mediating effect of family functionality was 0.082. Additionally, the chain-mediated effect, where marital and family functionalities interact, accounted for 0.063. The optimization of marital functionality significantly contributed to the improvement of family functionality (*r* = 0.063, *p* < 0.01). Based on these results, hypotheses H2, H2a, H2b, H2c, and H3 were tested.

**Table 6:** Decomposition of the path effect of sports participation on young people’s fertility intentions.

Influence pathways	Unstandardized path coefficients	*p*-value	Standardized coefficient	SE	CI-IB	CI-UB
Sports participation > Fertility intention	0.021	<0.05	0.025	0.011	0.003	0.046
Sports participation > Marital functioning	0.011	<0.01	0.032	0.011	0.009	0.055
Marital functioning > Fertility intention	0.0106	<0.1	0.023	0.016	0.008	0.054
Sports participation > Family functioning	0.007	<0.01	0.082	0.012	0.059	0.107
Marital functioning > Family functioning	0.017	<0.01	0.063	0.046	0.054	0.072
Family functioning > Fertility intention	0.059	<0.01	0.059	0.02	0.02	0.098

**Table 7:** Intermediary test results.

Intermediate path	Indirect effect size	SD	Ratio of indirect effects to total mediated effects	*p*-value	CI-IB	CI-UB
Sports participation > Marital functioning > Fertility intention	0.023	0.016	13.7%	<0.1	0.008	0.054
Sports participation > Family functioning > Fertility intention	0.082	0.012	48.8%	<0.01	0.059	0.107
Sports participation > Marital functioning > Family functioning > Fertility intention	0.063	0.046	37.5%	<0.01	0.054	0.072
Sports participation > Family systems functioning > Sports participation	0.168	0.050	1	<0.01	0.121	0.233

## 5 Discussion and analysis

The findings of this study, based on Self-Determination Theory (SDT), highlight the significant role of sports participation in shaping fertility intentions among young married individuals, with a notable increase in effect size when controlling for demographic and family-related variables. The baseline model (Model 1) revealed a statistically significant coefficient of 0.025 (*p* < 0.01), indicating that sports participation positively influences fertility intentions. Upon introducing control variables such as cohabitation segments, gender, age, age at first marriage, and number of family members in Model 2, the coefficient increased to 0.048 (*p* < 0.1), suggesting that the initial estimate may have been underestimated due to omitted variable bias.

While our models account for key demographic and family-related factors, we acknowledge the limitations in capturing other crucial determinants. For instance, the observed inverse association between educational attainment and fertility intentions may stem not only from prolonged schooling but also from stronger individualistic orientations and career-related apprehensions (21)—dimensions not quantified in the current framework. Similarly, the higher fertility rates prevalent in religious communities likely reflect doctrinal imperatives and faith-based support systems (22, 23) rather than purely socioeconomic considerations. Future investigations should incorporate validated metrics assessing three domains: (1) cultural paradigms (e.g., gender ideology, rural–urban value systems), (2) psychological well-being (e.g., depressive symptomatology, perceived stress), and (3) religious engagement (e.g., denominational affiliation, ritual frequency, congregational involvement). Adopting such a multidimensional analytic strategy, particularly through testing interaction effects like “educational attainment × individualistic orientation,” could elucidate how psychosocial mechanisms interface with lifestyle factors and familial dynamics to configure reproductive decision-making processes.

This enhanced effect size underscores the importance of considering contextual factors when examining the relationship between sports participation and fertility intentions. This pattern aligns with Greenhalgh’s (24) observation in rural China, where socioeconomic controls amplified the effects of physical activity on fertility intentions (24). This strengthened effect size is consistent with Western findings; for instance, Lois (25), using German pairfam data, reported a comparable impact of moderate exercise on childbearing intentions through stress reduction pathways (25). However, the marginal significance level (*p* < 0.1) warrants cautious interpretation. While physiological mechanisms (26) suggest benefits from exercise-induced hormonal regulation, endogeneity risks persist, as couples with higher fertility aspirations may self-select into fitness activities.

This study systematically examined the mediating role of family system functionality in the relationship between sports participation and fertility intentions using the Bootstrap method (1,000 resamples with bias correction, 95% confidence interval). The results revealed a total mediated effect of 0.168, indicating that sports participation influences fertility intentions through family system dynamics. This finding aligns closely with Billingsley’s report of a mediated effect based on European social survey data but significantly differs from Spracklen’s study of a Chinese cohort, which reported a higher mediated effect (27, 28). The discrepancy likely stems from differences in the conceptualization and measurement of family system functionality.

The decomposition of mediated effects revealed that marital subsystem functionality accounted for 0.023 of the indirect effect, while family subsystem functionality contributed 0.082. Additionally, a significant chain-mediated effect of 0.063 was observed, indicating that marital subsystem functionality enhances family subsystem functionality, which in turn influences fertility intentions. Although these mediation effects are statistically significant, their overall magnitude is relatively modest. Compared with empirical benchmarks—where some studies have demonstrated that community-based health interventions can improve correct reproductive health practices by approximately 35 percentage points (29)—our mediation pathway corresponds to roughly a 1–2% change in expected fertility outcomes. This suggests that, while sports participation holds promise as a component of pro-natal strategies, its standalone impact on aggregate fertility is likely limited in the short term and should be reinforced by complementary social policies. The positive correlation between marital subsystem functionality and family subsystem functionality (*r* = 0.063, *p* < 0.01) underscores the interdependence of these subsystems, consistent with Family Systems Theory (FST).

Comparative analysis with existing literature highlights several key points. First, the relatively low independent mediated effect of marital subsystem functionality (0.023) contrasts with findings from studies conducted in other East Asian countries, such as Japan and South Korea (30). This difference may reflect variations in cultural norms and family dynamics across regions. In China, where intergenerational influence remains significant, the decision-making process regarding fertility often involves extended family members, potentially diluting the direct impact of marital subsystem functionality.

Second, the chain-mediated effect (0.063) represents a substantial proportion (37.5%) of the total mediated effect, emphasizing the importance of understanding the dynamic interplay between marital and family subsystem functionalities. This finding supports Pinsof’s (31) “marital-family synergy model,” which posits that improvements in marital relationships can enhance broader family cohesion, ultimately influencing fertility intentions.

Cultural context plays a crucial role in interpreting these findings. In contrast to Western societies, where marital subsystem functionality often dominates the mediated effects, the effects in China are more diffused across both subsystems. This reflects the collectivist cultural context, where family harmony and intergenerational solidarity are highly valued, leading to a more integrated influence of both marital and family subsystem functionalities on fertility intentions.

Methodologically, this study advances previous research by employing a multilevel mediation model that accounts for the sequential and cumulative effects of marital and family subsystem functionalities. The use of bias-corrected Bootstrap methods provided robust estimates of the mediated effects, addressing limitations inherent in traditional Sobel tests.

In conclusion, this study offers valuable insights into the mechanisms underlying the relationship between sports participation and fertility intentions. By integrating theoretical perspectives from Self-Determination Theory and Family Systems Theory, this research not only enhances our understanding of fertility decision-making but also provides practical implications for designing interventions aimed at improving family well-being and promoting reproductive health.

## 6 Conclusions, recommendations, limitations

### 6.1 Conclusion

By organizing the CFPS2022 data and constructing the SDT-FST theoretical framework, this study finds that sports participation has a significant positive effect on the fertility intentions of married youth. Rather than acting solely through direct influence, sports participation contributes to improved marital and family functioning, which together form a sequential pathway linking physical activity to fertility-related decision-making. From the SDT perspective, participation in sports may satisfy key psychological needs—autonomy, competence, and relatedness—thereby improving individuals’ intrinsic motivation and well-being. At the same time, the results support FST by showing how an activity outside the home can positively influence intra-family relationships. Improved marital functioning appears to set off a ripple effect that strengthens the entire family system, making couples more confident and prepared to expand their family.

It is also noteworthy that these psychological and relational mechanisms are embedded in China’s cultural context of strong familial ties and intergenerational support. In a collectivist society like China, a harmonious marriage and well-functioning family not only benefit the couple but also signal stability to the extended family. Sports participation can thus act as a modern conduit to reinforce traditional family values: when young couples engage regularly in sports, they tend to experience greater marital harmony and family cohesion, which may encourage parents and in-laws to offer more support with childcare. Such intergenerational support, in turn, further eases young couples’ concerns about raising children. By bridging SDT and FST within the Chinese cultural setting, this study provides a more comprehensive understanding of how lifestyle behaviors like sports engagement intertwine with personal motivations and family dynamics to shape married youths’ fertility intentions.

### 6.2 Recommendation

First, promoting sports activities: Governments and social organizations should strengthen the construction of sports facilities and the promotion of activities, especially among youth groups. For example, they should set up more fitness facilities and activity programs in communities or workplaces and encourage young people to actively participate in sports activities. This will not only improve their physical health, but also enhance the fulfillment of their psychological needs, which will in turn increase their willingness to give birth.

Second, formulate family-friendly policies: Taking into account the results of the study, it is recommended that family-friendly policies such as extending maternity leave and providing childcare subsidies be formulated. These policies can reduce the pressure on young families in giving birth and raising children, thus enhancing their willingness to give birth. For example, providing parental leave and financial support to enable young parents to better balance work and family responsibilities.

Third, health promotion campaigns: Through health promotion campaigns, emphasize the positive impact of physical activity on physical and mental health and fertility. For example, promotional materials could highlight how physical activity enhances reproductive health, strengthens family cohesion, and promotes fertility intentions. This will change public perceptions and attitudes toward physical activity and encourage more young people to participate.

Fourth, local governments and social organizations can address declining fertility rates through five synergistic strategies: (1) hosting regular “Family Fitness Days” in public spaces—featuring parent–child walks, yoga sessions, and recreational sports—to strengthen intergenerational bonds while fostering exercise habits; (2) offering fitness vouchers and equipment subsidies to families enrolled in community programs, effectively reducing economic barriers to participation; (3) designing intergenerational initiatives like grandparent-grandchild sports leagues and family dance festivals that build shared memories and reinforce caregiving network; (4) extending gender-neutral paid parental leave beyond existing maternity frameworks while subsidizing childcare services to alleviate work–family conflicts; and (5) deploying multimedia health campaigns with resonant slogans (e.g., “Active Bodies, Thriving Families”) paired with authentic family narratives, demonstrating how physical wellness contributes to household harmony—a crucial psychosocial pathway for indirectly nurturing fertility aspirations.

### 6.3 Limitation

First, the limitations of the cross-sectional design: this study employs a cross-sectional design, which can only identify correlations between variables and does not establish causal relationships. For example, it remains unclear whether sports participation leads to increased fertility intentions, or whether individuals with higher fertility intentions are more likely to engage in sports. This limitation restricts our understanding of causality.

Second, in this study, fertility intention was assessed using a single binary (yes/no) item, which may introduce measurement bias or misclassification. Specifically, this approach cannot distinguish between variations in the strength of respondents’ childbearing desire and their preferred timing. Future research should consider employing multi-point scales or continuous measures—ideally complemented by qualitative interviews—to enhance the precision and validity of this construct.

Third, the absence of longitudinal data: without tracking data, the long-term effects of sports participation on fertility intentions cannot be observed. Additionally, the dynamic changes in intermediate variables cannot be captured, limiting our understanding of the underlying mechanisms.

Fourth, the impact of social change: the results of the study may be influenced by broader social changes. For instance, factors such as economic development and shifts in fertility policies could alter the relationship between sports participation and fertility intentions. If these external factors are not accounted for, the robustness of the findings may be compromised.

## Data Availability

The original contributions presented in the study are included in the article/supplementary material, further inquiries can be directed to the corresponding author.
